# Similarity-weighted entropy for quantifying genetic diversity in viral quasispecies

**DOI:** 10.1093/ve/veaf029

**Published:** 2025-04-26

**Authors:** Jian Wu

**Affiliations:** State Key Laboratory for Managing Biotic and Chemical Threats to the Quality and Safety of Agro-products, Key Laboratory of Biotechnology in Plant Protection of MARA, Key Laboratory of Green Plant Protection of Zhejiang Province, Institute of Plant Virology, Ningbo University, Ningbo 315211, China

**Keywords:** Viral quasispecies, genetic diversity, entropy, sequence similarity

## Abstract

A viral quasispecies is a genetically diverse population of closely related viral variants that exist in a state of dynamic equilibrium. This diversity, driven by mutations, recombination, and selective pressures, enables viruses to adapt rapidly, affecting pathogenicity and treatment resistance. Quantifying the genetic diversity within viral quasispecies is therefore crucial for understanding viral evolution and for designing effective therapeutic strategies. Entropy is a commonly used metric to measure genetic diversity within such populations; however, traditional entropy calculations often neglect genetic similarities between sequences, which can result in overestimating true diversity. In this study, I compare several widely used diversity indices for quantifying viral quasispecies diversity and introduce a novel similarity-weighted entropy metric that incorporates sequence similarity into entropy calculations. This approach enables a more comprehensive representation of diversity in genetically cohesive viral populations. By applying both conventional and similarity-weighted entropy calculations to hypothetical sequence populations and real viroid and virus quasispecies, I demonstrate that similarity-weighted entropy provides a more comprehensive measure of genetic diversity while maintaining the simplicity of conventional entropy. These findings highlight the value of similarity-weighted entropy in characterizing viral quasispecies and its potential to improve our understanding of viral adaptation and resistance mechanisms

## 1. Introduction

Viral quasispecies, originally proposed by Eigen in 1971 (Eigen 1971), represent a population of related viral sequences within an infected host (Domingo and Perales 2019). Due to the error-prone nature of viral replication, especially in RNA viruses and viroids, a high degree of genetic diversity exists among these sequences (Gago et al. 2009, Wu et al. 2020a, 2020b, Jones et al. 2021, Wu and Bisaro 2024a). This diversity allows viral quasispecies to adapt rapidly to changing environments, evade immune responses, and develop resistance to antiviral treatments (Domingo et al. 2021).

Quantifying genetic diversity is essential for understanding the behaviour and evolution of quasispecies (Smith et al. 1997, Smerlak 2021). Shannon entropy and normalized entropy are commonly used to describe the genetic heterogeneity of a population, with higher entropy reflecting greater diversity (Arbiza et al. 2010, Gregori et al. 2016, 2023, Wu et al. 2024). However, traditional entropy measures do not take into account the genetic similarity between sequences. In populations where sequences differ by only a few mutations, entropy may overestimate the effective diversity by treating similar sequences as entirely distinct. This limitation calls for an improved metric that incorporates sequence similarity into entropy calculations. Weighted entropy is a variation of the standard Shannon entropy, where different elements in a dataset contribute differently based on assigned weights (Guiaşu 1971, Kelbert et al. 2017, Mahdy 2018). In a biological or genetic context, weighted entropy can be used to measure the diversity or uncertainty of sequences while accounting for the importance or similarity of individual sequences (Chang and Wang 2011, Xie et al. 2024). However, the use of weighted entropy to analyse the genetic diversity of viral quasispecies based on sequence similarity has not yet been reported.

Common indices for assessing viral quasispecies diversity include minimum mutation frequency (Mf min), maximum mutation frequency (Mf max), normalized Shannon entropy (*H_n_*), and nucleotide diversity (*π*). Mf min indicates the proportion of positions with unique mutations across sequence reads, counting each mutation only once per genomic position across different clones. In contrast, Mf max reflects the proportion of positions with mutations in the sequence set, with mutations identified by comparing each sequence to the population consensus sequence. Shannon entropy (*H*) captures diversity based on haplotype frequencies, commonly normalized to log_2_(*T*), where *T* represents the total number of clones; alternatively, it may be normalized to log_2_(*N*), where *N* is the estimated number of unique sequences, following ecological study practices. Normalization to log_2_(*N*) is particularly common in next generation sequencing (NGS) data analyses. Mean pairwise divergence measures the average genetic distance between all pairs of sequences. This distance can be measured using various methods, such as the number of nucleotide differences, the Hamming distance (which counts mismatches), or other more sophisticated distance metrics like Kimura’s two-parameter distance. Mean pairwise divergence offers richer insights than Mf by considering the diversity across all genome pairs in the population (Gregori et al. 2016). However, mean pairwise divergence does not capture changes in the dynamic distribution of sequence frequencies within the population. Together, these four indices primarily assess the frequency of unique sequences and sequence dissimilarity within the population. However, no single index simultaneously measures both aspects.

**Figure 1.**
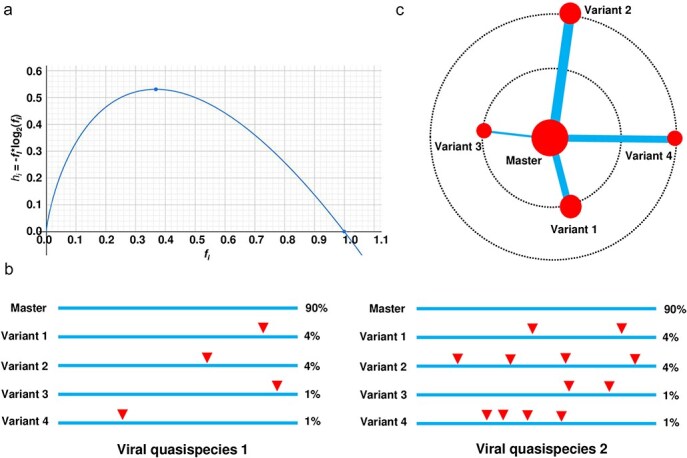
**Rationale for including sequence similarity as a weight in entropy calculations to reflect genetic diversity in viral quasispecies**. (a) The curve for *h_i_* = −f_I_*log_2_(*f_i_*) illustrates how entropy is influenced by the frequency of each unique sequence *i* (*f_i_*) in a population. The entropy *H* is the sum of the individual entropies *hi*. The peak occurs at approximately *f_i_* = 1/e ≈ 0.3679 and decreases as *f_i_* approaches either 0 or 1. The plot helps visualize the balance between diversity (represented by nonzero frequencies across many unique sequences) and uniformity (where high frequencies for a few sequences reduce entropy). (b) Analysing sequence similarity in two hypothetical viral quasispecies reveals limitations of traditional entropy in capturing genetic diversity. Standard entropy metrics focus on the distribution of variant frequencies, disregarding genetic relationships between sequences. In both hypothetical quasispecies 1 and 2, a master sequence accounts for 90% of the population, with four variants making up 4%, 4%, 1%, and 1%, respectively. This distribution yields identical entropy-based diversity values for both quasispecies. However, in reality, the variants in quasispecies 2 contain more mutations, indicated by the red solid triangles, resulting in a higher actual genetic diversity compared to quasispecies 1. (c) The principle of incorporating sequence similarity as a weight in entropy calculation can be illustrated using a master sequence, represented by the largest red solid circle in the centre. This diagram helps to explain the relationship between sequence diversity, genetic distance (sequence similarity), and weighting in the calculation. Viral quasispecies 2 presented in (b) are used as an example. Two dashed circles indicate levels of genetic distance: the larger dashed circle represents greater distance, while the smaller one indicates closer similarity. The size of each red circle corresponds to sequence frequency. The thickness of lines connecting the master sequence to its variants represents the weights added to entropy, with greater genetic distance and higher variant frequency contributing more to genetic diversity.

To more comprehensively quantify genetic diversity in viral quasispecies, I examined the rationale for including sequence similarity as a weighting factor in entropy calculations. Entropy is traditionally calculated as *h_i_* =−*f_i_*⋅log_2_(*f_i_*), where *fi* represents the frequency of each unique sequence *i* in a population. This function reaches its peak at approximately *f_i_* =1/e ≈ 0.3679 and decreases as *f_i_* approaches either 0 or 1 (Fig. 1a), highlighting the balance between high diversity (nonzero frequencies across many unique sequences) and uniformity (dominated by a few high-frequency sequences). The entropy *H* is the sum of the individual entropies *h_i_*. This curve illustrates how standard entropy reflects frequency distribution but may overlook significant aspects of genetic diversity when sequence similarity is not considered. In this study, I introduce a similarity-weighted entropy metric that incorporates sequence similarity into entropy calculations, aiming to overcome the limitations of traditional entropy in capturing diversity within viral quasispecies. I apply this new metric alongside conventional entropy to both simulated sequence populations and real viroid and virus quasispecies populations, comparing the results to demonstrate the advantages of similarity-weighted entropy. Our findings underscore the potential of this approach to provide a more comprehensive representation of genetic diversity in highly similar viral populations, enhancing our understanding of viral adaptation and resilience.

## 2. Methods

### 2.1 Formulas for traditional entropy and similarity-weighted entropy calculations

Traditional entropy (*H*):


$$H = - \sum\limits_i^{} {} {f_i} \cdot {\log _2}({f_i})$$

where *f_i_* is the frequency of each unique sequence *i* in the population.

Normalized entropy (*H_n_*):


$${H_n} = \frac{{ - {\sum _i}{f_i} \cdot {{\log }_2}({f_i})}}{{{{\log }_{_2}}(N)}}$$

where *N* is the total number of unique sequences in the population. This normalization allows *H_n_* to range between 0 and 1.

Similarity-weighted entropy (*H*_sim_):


$${H_{sim}} = - \sum\limits_i^{} {} {f_i} \cdot {\log _2}({f_i}) \cdot (1 - {S_{mean,weighted}}(i))$$

$$S_{mean,weighted}(i)=\sum_{i=\!\!\!\!/i}^{}\,\,f_i\,\,s_{ij},$$

where *S_ij_* represents the sequence similarity between sequences *i* and *j*. This term adjusts each sequence’s contribution to entropy based on its similarity to others. In this study, both simulated and real quasispecies populations consist of sequences with identical lengths within each population, but different populations may vary in sequence length. Sequences within a population differ solely by base substitutions, with no insertions or deletions. The pairwise similarity metric, *S_ij_*, is defined as the number of base substitutions between sequences *i* and *j*, normalized by the sequence length.

Normalized similarity-weighted entropy (*H_n_*_sim_):


$$H = - \sum\limits_i^{} {} {f_i} \cdot {\log _2}({f_i})$$

Similar to *H_n_*, this metric normalizes *H*_sim_ by log_2_(*T*) to allow for values between 0 and 1.

The rationale behind the formulas for *H*_sim_ and *H_n_*_sim_ is detailed in the Results section.

### 2.2 Deep sequencing analysis of tomato brown rugose fruit virus quasispecies

Tomato plants carrying either the *Tm-2^2^* resistant allele (cultivar ‘Jinpeng No. 1ʹ) or the *tm-2* susceptible allele (cultivar ‘Rutgers’) were infected with tomato brown rugose fruit virus (ToBRFV) via agro-infiltration using a plasmid containing ToBRFV DNA. Single cells were harvested from the youngest leaves at 15 days postinfection, and sequencing was conducted by Kidio (Guangzhou, China) using the 10× Genomics Chromium system, which generated 100 GB of data per sample. ToBRFV reads were extracted from the datasets using the ToBRFV reference genome (GenBank ID: MT018320.1) by Kidio. Reads covering the first 120 nucleotides of the 5ʹ end of ToBRFV were then further selected for subsequent analysis.

### 2.3 Computational analysis

Computational analysis was performed using a custom Java program with three primary functions. The Similarity Matrix Generator function generated similarity matrices (*S_ij_*) for sequence populations, while the Entropy Calculator function calculated values for *H, H_n_, H*_sim_, and *H_n_*_sim_. The SequenceGeneratorN function produced populations with saturated mutations for sequences ranging in length from a single nucleotide (N) to seven nucleotides (7Ns). Sequencing data for the PSTVd loop 27 mutant pool were sourced from my previous study (Wu et al. 2024). The Hamming Distance Matrix Calculator was used to generate a distance matrix and calculate the average Hamming distance. The Java code has been uploaded to Github (https://github.com/tomwu1495/Similarity-Weighted-Entropy).

## 3. Results

### 3.1 Evaluation of traditional and the new entropy metrics using multiple types of simulated datasets

Two hypothetical viral quasispecies were analysed for genetic diversity using traditional entropy-based metrics (Fig. 1b). In both quasispecies, a master sequence comprises 90% of the population, with four variants at frequencies of 4%, 4%, 1%, and 1%, resulting in identical entropy values. However, quasispecies 2 contains more distant variants due to additional mutations (shown as red triangles), leading to greater actual genetic diversity. This illustrates that traditional entropy metrics fail to account for genetic relationships between sequences, potentially underestimating diversity. To address this, I proposed two similarity-weighted entropy metrics (*H_n_* and *H_n_*_sim_, see Methods), incorporating sequence similarity into the entropy calculation. Both metrics assigns weights based on genetic distance from the master sequence, providing a more comprehensive measure of diversity. A diagram of quasispecies 2 (Fig. 1c) shows the master sequence as the largest red circle, with lines indicating genetic distance. Larger circles reflect higher frequencies, and thicker lines indicate greater similarity weights, emphasizing both frequency and genetic distance in quantifying diversity.

**Figure 2.**
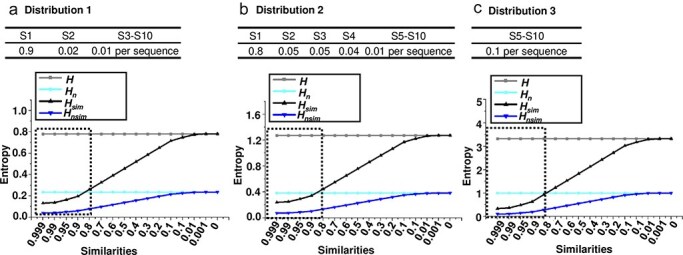
**Limitations of conventional diversity indices in analysing viral quasispecies with similar variant frequencies but different sequence similarities**. Four entropy-based metrics—*H*, *H_n_*, *H*_sim_, and *H_n_*_sim_—were used to evaluate their effectiveness in describing viral quasispecies diversity across varying sequence similarity levels. Viral quasispecies typically consist of a dominant master sequence with medium- to low-frequency variants that share high sequence similarity, with values typically above 80%, as lower similarity could indicate the emergence of distinct viral species. To simulate realistic quasispecies populations, three distributions of 10 sequences with different frequency profiles were designed: (a) Distribution 1, which includes a high-frequency master sequence, a few medium-frequency variants, and several low-frequency variants, reflecting a common structure in viral quasispecies; (b) Distribution 2, similar to Distribution 1, but with the master sequence at a slightly lower frequency and the remaining frequencies more evenly distributed among the medium- and low-frequency variants, mimicking viral populations with a more even variant diversity; and (c) Distribution 3, a uniform distribution in which all variants have equal frequency, representing a maximally diverse population without a dominant master sequence. For each distribution, *H*, *H_n_*, *H*_sim_, and *H_n_*_sim_ were calculated across a range of sequence similarity values (from 0.999 to 0) on the *x*-axis, with sequence frequencies (S1–S10) also presented. Dashed-line rectangles indicated the sequence similarity values typically observed within viral quasispecies.

**Figure 3.**
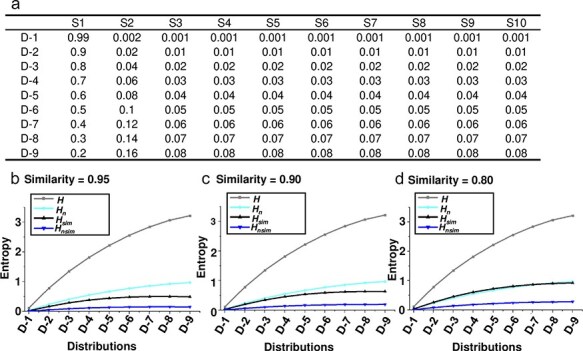
**Entropy metrics across varying sequence distributions at different fixed sequence similarities**. Four entropy calculation methods (*H*, *H_n_*, *H*_sim_, and *H_n_*_sim_) were tested across nine distributions from D-1 to D-9 (a) at fixed sequence similarities of 0.95 (b), 0.90 (c), and 0.80 (d). On the *x*-axis, the sequence distributions ranged from D-1, where a single dominant sequence (master) prevails in the population, to D-9, where all sequences have more balanced and higher frequencies, creating a more diverse population.

**Figure 4.**
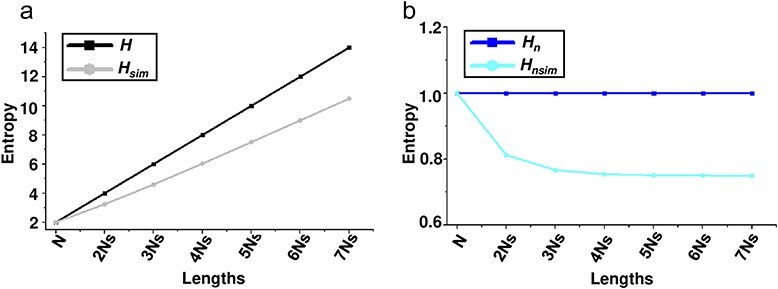
**Analysis of four entropy metrics (*H*, *H_n_*, *H*_sim_, and *H_n_*_sim_) using sequence populations with saturated mutations and uniform sequence frequencies**. Sequence populations were generated with fully saturated mutations for N (4 sequences), NN (16 sequences), NNN (64 sequences), up to 7 Ns (16,384 sequences), all with equal sequence frequencies, forming uniform distributions. For clarity, *H* and *H_n_* are presented in panel (a), while *H*_sim_ and *H_n_*_sim_ are displayed in panel (b). *X*-axis represents the lengths of sequences in each population.

To better understand the performance of the four entropy metrics (*H, H_n_, H*_sim_, and *H_n_*_sim_, see Methods) in different contexts, multiple simulated datasets were generated, each containing viral quasispecies with varying sequence distributions, frequencies, similarities, and numbers of unique sequences. I first analysed quasispecies populations with identical frequencies but varying sequence similarity. Three distributions were simulated: (i) a dominant master sequence with a few low-frequency variants (Distribution 1), representing a typical quasispecies structure; (ii) a more balanced distribution (Distribution 2); and (iii) a uniform distribution (Distribution 3), with equal frequencies for all variants (Fig. 2a–c). All four metrics increased from Distribution 1 to Distribution 3, indicating their sensitivity to changes in sequence distribution. While *H* and *H_n_* remained unchanged across all similarity levels, *H*_sim_ and *H_n_*_sim_ increased as sequence similarity decreased. Next, I tested the metrics with populations exhibiting different frequency distributions (Fig. 3a) but fixed sequence similarity at 0.95 (Fig. 3b), 0.90 (Fig. 3c), and 0.80 (Fig. 3d). As the population became more diverse (from D-1 to D-9), the entropy values for *H* and *H_n_* increased, reflecting a greater spread of variant frequencies. However, *H*_sim_ and *H_n_*_sim_ showed a more gradual increase, since they also account for sequence similarity. Finally, I analysed populations with saturated mutations, a common approach to studying viral quasispecies evolution (Wu et al. 2024). Sequence length was varied from 1 to 7 nucleotides. As sequence length grew, *H*_sim_ increased more gradually compared to *H*, which showed a sharp rise with each additional nucleotide (Fig. 4a). Meanwhile, *H_n_* remained constant at its maximum value of 1, while *H_n_*_sim_ gradually decreased as sequence similarity inevitably accumulated with increasing sequence length (Fig. 4b).

These findings highlight the limitations of *H* and *H_n_* in accounting for sequence similarity and emphasize the unique distinctions captured by *H*_sim_ and *H_n_*_sim_, particularly in populations that are diverse yet internally similar.

It is worth noting that additional populations, varying in the number of unique sequences, sequence distributions, and sequence similarities, were also tested, yielding similar results (Supplementary File 1).

### 3.2 Analysis of a real viroid quasispecies demonstrated the advantage of similarity-weighted entropy indices in capturing functional diversity

**Figure 5.**
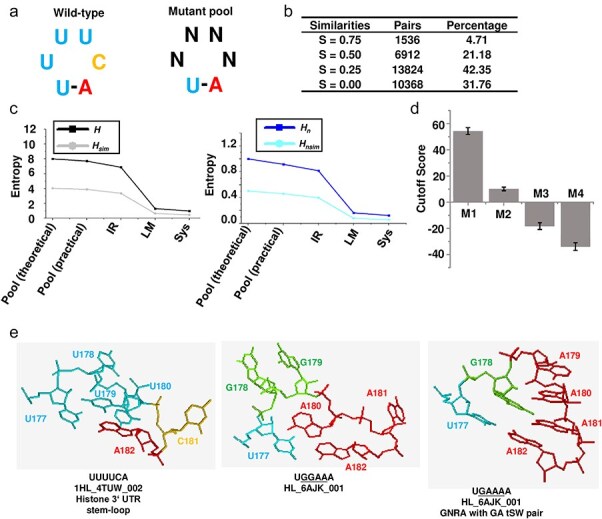
**Analysis of a real viroid quasispecies demonstrated the advantage of similarity-weighted entropy indices in capturing functional diversity**. (a) Schematic representation of the mutagenesis strategy applied to the PSTVd loop 27 region, spanning nucleotides 177–182. The wild-type sequence, UUUUCA, forms a hairpin structure with a four-nucleotide loop (UUUC) and a UA base pair that closes the loop. The wild-type sequence was mutated to UNNNNA, where each N represents one of four nucleotides (A, U, G, or C), generating 256 unique sequence variants. (b) A pairwise similarity matrix for the 256 variants was generated and summarized, showing those similarity values of 0.75, 0.50, 0.25, and 0.00 account for 4.71%, 21.18%, 0.25%, and 0.00% of all pairs, respectively. (c) Comparison of entropy metrics (*H, H_n_, H*_sim_, and *H_n_*_sim_) across the theoretical mutant pool (with equal frequencies for all 256 sequences), the practical pool generated by saturated mutagenesis, and the evolved pool after replication in the inoculated region, migration to leaf margins (LM), and trafficking to systemic leaves (Sys samples) within the plant. (d) Structural compatibility of each mutant sequence relative to the wild-type PSTVd loop 27, grouped by mismatch count (M1–M4). The JAR3D tool (https://rna.bgsu.edu/jar3d/) was utilized to align the mutant sequences with the 3D structural model of the wild-type PSTVd loop 27 (UUUCA). The resulting Cutoff Score reflects the compatibility between each mutant sequence and the wild-type model. Higher Cutoff Scores indicate stronger structural similarity to the wild-type, and a decrease in score correlates with an increase in mismatches. The Cutoff Scores were presented as the mean ± standard error. (e) Structural comparison of selected mutants (UGGAAA and UGAAAA) and the wild-type sequence (UUUCA). JAR3D was used to predict the structural models for both wild-type and mutant sequences. Models with identical sequences were identified. Mutants UGGAAA and UGAAAA form distinct structures from the wild-type, potentially indicating functional diversification.

In a previous study, I investigated the role of RNA 3D structure in constraining the sequence diversity of potato spindle tuber viroid (PSTVd) quasispecies (Wu et al. 2024). One example is the sequence of loop 27, which spans nucleotides 177–182 and is composed of the sequence UUUUCA. The 3D structure of loop 27 plays a critical role in mediating the unidirectional movement of PSTVd from epidermal cells to mesophyll cells. Loss of this structure impairs the local infection of PSTVd (Wu et al. 2024, Wu and Bisaro 2024b). This loop is closed by a UA base pair, forming a structure with four nucleotides. To explore the effects of sequence variation in this region, I performed saturated mutagenesis on the loop (with the sequence UNNNNA, where N represents any of the four nucleotides: A, U, G, or C). The resulting mutant pool consisted of 256 distinct variants, representing all possible sequence combinations for this loop (Fig. 5a). Analysis of the pairwise similarity matrix for the 256 mutants revealed the following distribution of sequence similarities: 1536 pairs (4.71%) had a similarity of 0.75; 6912 pairs (21.18%) had a similarity of 0.50; 13 824 pairs (42.35%) had a similarity of 0.25; and 10 368 pairs (31.76%) had a similarity of 0.00 (Fig. 5b). The values of *H, H_n_, H*_sim_, and *H_n_*_sim_ were calculated. For clarity, *H* and *H*_sim_, as well as *H_n_* and *H_n_*_sim_, were presented separately.

In theory, the pool should consist of 4^4^ = 256 sequences, each with an identical frequency (Theoretical pool). Therefore, the value of *H* should be 8, and the value of *H*_sim_ should be 4.04 (Fig. 5c). For the Theoretical pool, the value of *H_n_* is 1, while the value of *H_n_*_sim_ is 0.50. This indicates that the sequence similarity in this pool (Fig. 5b) reduces the value of *H*_sim_ and *H_n_*_sim_. In practice, the values for the practical pool closely approximate the theoretical values, suggesting that the mutant pool was successfully prepared. After replication in the inoculated region (IR samples), migration to the margin of the inoculated leaves (LM samples), and trafficking to systemic leaves (Sys samples), all four values gradually decreased. Notably, the *H* value decreases more sharply than *H*_sim_, and the *H_n_* value decreases more sharply than *H_n_*_sim_.

Although analysing the function of all 256 mutants is not feasible, the principle that RNA 3D structure determines RNA function suggests that examining the structural diversity of these mutants can provide insights into functional and genetic diversity. To investigate this, I compared the 255 mutants to the wild-type sequence (UUUCA) and categorized them into four groups based on the number of mismatches: M1, M2, M3, and M4, corresponding to 1, 2, 3, and 4 mismatches, respectively. The JAR3D tool (https://rna.bgsu.edu/jar3d/) was used to align the mutant sequences with the 3D structural model of wild-type PSTVd loop 27 (UUUUCA), which is the loop region of 3ʹ stem-loop of animal histone mRNA (Wu et al. 2024). The resulting Cutoff Score reflects the compatibility between each mutant sequence and the wild-type model, with a higher Cutoff Score indicating greater compatibility. As expected, I observed that as the number of mismatches increased, the compatibility decreased significantly, suggesting that reduced sequence similarity could lead to the formation of novel RNA structures with distinct functional properties (Fig. 5d). In fact, when comparing the structural models of specific mutants, such as UGGAAA and UGAAAA, I observed that these mutants adopt distinct structures compared to the wild-type. Notably, UGAAAA forms a GNRA-type structure with a GA tSW pair, indicating that this mutant loop possesses a markedly different structure and potentially a different function than the wild-type (Fig. 5e). These results suggest that introducing sequences with lower similarity to the master sequence into viral quasispecies may substantially increase genetic diversity by promoting the emergence of new functional RNA structures. In contrast, sequences with high similarity to the wild-type may contribute less to genetic diversity. To fully capture the contribution of these sequence types to the overall genetic diversity of viral quasispecies, entropy calculations must be weighted by sequence similarity. This approach would provide a more comprehensive measure of how variations in sequence similarity influence the genetic diversity of the viral population.

### 3.3 Similarity-weighted entropy revealed the increased sequence diversity of ToBRFV induced by *Tm-2^2^* anti-viral gene

**Figure 6.**
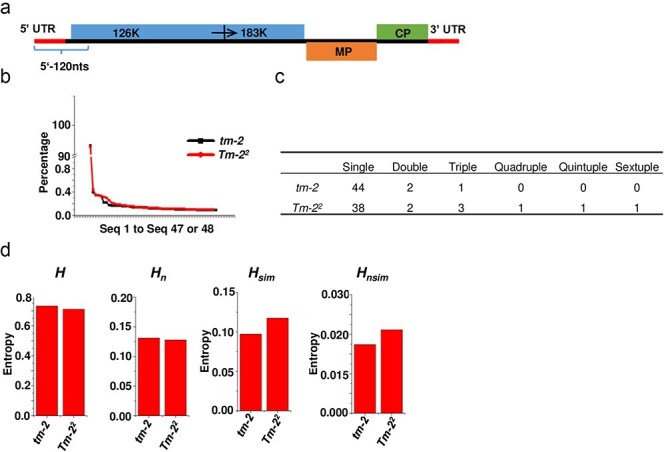
**Sequence diversity of ToBRFV in *tm-2* and *Tm-2^2^* plants revealed by four entropy metrics**. (a) Genome organization of ToBRFV. ToBRFV encodes two key proteins for RNA replication: a 126 kDa protein and a 183 kDa protein, the latter produced via ribosomal readthrough of the 126 kDa protein’s termination codon. ToBRFV also encodes a MP and a CP. The 5′ UTR acts as a translational enhancer, while the 3′ UTR increases mRNA stability. This study focuses on 73 nucleotides (nts) of the 5′ UTR and the first 47 nts of the gene encoding the 126 kDa protein, a region totalling 120 nts, which I named 5′-120nts. (b) Variants of the 5′-120nts region in ToBRFV-infected *tm-2* and *Tm-2^2^* plants were sequenced using single-cell RNA sequencing performed with the 10× Genomics Chromium system. Unique sequences were identified, and the number of reads for each sequence was counted. The abundance (percentage) of each unique sequence was then calculated. To exclude errors introduced during library preparation and sequencing, sequences with an abundance below 0.1% of the total reads were excluded. As a result, a total of 48 and 47 unique sequences were identified for the *tm-2* and *Tm-2^2^* samples, respectively. The abundance of each unique sequence in the two samples is presented. (c) The sequences of each unique variant were compared to the master sequence (Seq1), and the variants were classified based on the number of mutations. Variants were categorized as single, double, triple, quadruple, quintuple, and sextuple mutants. (d) *H*, *H_n_*, *H*_sim_, and *H_n_*_sim_ were calculated for the quasispecies of the 5′-120nts region identified from the *tm-2* and *Tm-2^2^* samples. These values were then compared between the two samples.

The quasispecies of a plant virus named ToBRFV was used to further assess these four metrics. ToBRFV encodes two essential proteins for RNA replication: a 126 kDa protein and a 183 kDa protein, the latter of which is generated through ribosomal readthrough of the 126 kDa protein’s stop codon. Additionally, ToBRFV encodes a movement protein (MP) and a capsid protein (CP). The 5ʹ untranslated region (UTR) serves as a translational enhancer, while the 3ʹ UTR plays a role in stabilizing the mRNA (Fig. 6a). The tomato *Tm-2^2^* gene is recognized as one of the most durable resistance genes, yet ToBRFV has successfully overcome this resistance. In this study, the quasispecies of the first 120 nucleotides at the 5ʹ end of ToBRFV (named 5ʹ-120nts) was extracted from single-cell RNA sequencing datasets of ToBRFV-infected tomato plants carrying either the *Tm-2^2^* resistant allele or the *tm-2* susceptible allele. The focus on this region is due to a higher number of reads being mapped to the 5ʹ end of ToBRFV compared to other regions, with read lengths typically ranging between 120 and 150 nucleotides. Unique sequences were identified, and the read count for each sequence was determined. The abundance (percentage) of each unique sequence was then calculated. To minimize errors introduced during library preparation and sequencing, sequences with an abundance below 0.1% of the total reads were excluded. Consequently, 48 unique sequences were identified in the *tm-2* sample, and 47 unique sequences were identified in the *Tm-2^2^* sample. Detailed information on the unique sequences was presented in Supplementary File 2. The abundance of each unique sequence in both samples is presented. Both populations contain a dominant master sequence, which accounts for approximately 93% of the total sequences. However, the distribution of minor variants differs slightly between the two populations (Fig. 6b). The sequences of each unique variant were compared to the master sequence (Seq1) to assess the genetic divergence within the populations. Only base substitutions, but not insertions and deletions, were detected. Based on the number of mutations relative to the master sequence, the variants were classified into distinct categories: single, double, triple, quadruple, quintuple, and sextuple mutants. Interestingly, variants from the *Tm-2^2^* sample exhibited a greater accumulation of mutations compared to those from the *tm-2* sample (Fig. 6c). This suggests that the *Tm-2^2^* allele may drive a more diverse mutation profile in response to ToBRFV infection, potentially reflecting a stronger selective pressure or a more dynamic quasispecies evolution compared to the *tm-2* allele, which is susceptible to the virus. The Hamming distance between pairs of sequences from both populations was calculated and presented in a matrix (Supplementary File 3). In the *tm-2* sample, the Hamming distance ranged from 0 to 5, whereas in the *Tm-2^2^* sample, it ranged from 0 to 10. The average Hamming distance was 2.12 for the *tm-2* sample and 2.80 for the *Tm-2^2^* sample. This indicates a higher level of genetic divergence in the *Tm-2^2^* sample, suggesting that the *Tm-2^2^* allele may drive a more diverse quasispecies population in response to ToBRFV infection compared to the *tm-2* sample. *H, H_n_, H*_sim_, and *H_n_*_sim_ were also calculated for the quasispecies of the 5′-120nts region identified from the *tm-2* and *Tm-2^2^* samples. *H* and *H_n_* are slightly higher in *tm-2* sample compared to *Tm-2^2^* sample, while *H*_sim_, and *H_n_*_sim_ values are much higher in *Tm-2^2^* sample than in *tm-2* sample.

Although Hamming distance is commonly used alongside traditional Shannon entropy to assess genetic diversity in viral quasispecies, the results from these two metrics in this study were contradictory. The Hamming distance indicated higher genetic diversity in the *Tm-2^2^* sample, while traditional Shannon entropy suggested the opposite. This discrepancy makes it difficult to draw definitive conclusions based on these two values alone. In contrast, the similarity-weighted entropy metrics, *H*_sim_ and *H_n_*_sim_, which incorporate both sequence distribution and sequence similarity, provided a clearer picture of the genetic diversity. These metrics revealed the strong selection pressure exerted by the *Tm-2^2^* allele on ToBRFV, highlighting the utility of similarity-weighted entropy in more accurately capturing viral quasispecies dynamics.

## 4. Discussion

The primary aim of this study was to assess the performance of different metrics—Shannon entropy (*H*), normalized Shannon entropy (*H_n_*), similarity-weighted entropy (*H*_sim_), similarity-weighted normalized entropy (*H_n_*_sim_), and other indices, such as mean pairwise divergence—in quantifying the genetic diversity of viral quasispecies. The results demonstrate that while traditional entropy measures (*H* and *H_n_*) remain widely used, they fall short of comprehensively capturing the complexity of diversity in viral populations with high sequence similarity. In contrast, the newly introduced similarity-weighted entropy indices (*H*_sim_ and *H_n_*_sim_) provide a more nuanced understanding by incorporating both sequence similarity and frequency distribution, offering a better reflection of viral population structure, genetic diversity, and potential for adaptation. These metrics can be compared to Earth-Mover’s Distance (EMD) and Symmetrized Kullback–Leibler Divergence (Jensen–Shannon Divergence, JSD), which are commonly used in population genetics (Düsterhus and Andreas 2012). EMD quantifies the effort to transform one distribution into another, considering both frequency and genetic distance. While my entropy metrics do not directly calculate EMD, they share a similar goal of quantifying distributional differences by incorporating sequence similarity. JSD, a symmetrized version of Kullback–Leibler divergence, compares two distributions and measures their divergence. My metrics, in contrast, focus on measuring diversity within a single population, integrating both frequency and similarity, rather than comparing two separate distributions. A key advantage of similarity-weighted entropy is that it efficiently captures within-population diversity without requiring pairwise comparisons, making it more suitable for ranking or comparing multiple populations. For better comparison between my metrics and traditional ones, this discussion covers the traditional entropy metrics, the new similarity-weighted entropy metrics, and the widely used mean pairwise divergence.

### 4.1 Traditional entropy metrics (*H* and *H_n_*): limited sensitivity to genetic similarity

As demonstrated in Figs 2 and 3, *H* and its normalized form *H_n_* are sensitive only to the frequency distribution of variants, and not to the genetic similarity between them. In populations where a dominant master sequence coexists with a few low-frequency variants (as shown in Distribution 1 and Distribution 2), *H* and *H_n_* fail to capture the genetic distance between highly similar sequences. For example, when the sequence similarity within a quasispecies increased (approaching 100%), these metrics remained unchanged, even though the sequences became more genetically similar (Fig. 2). This indicates that *H* and *H_n_* do not reflect important aspects of diversity that stem from the genetic relationships between the sequences themselves.

The inability of *H* and *H_n_* to incorporate sequence similarity becomes particularly problematic in viral quasispecies, where variants often differ by only a few mutations (Domingo et al. 2012; Aparicio et al. 2022). This limitation means that *H* and *H_n_* can potentially overestimate the diversity of a viral population, treating very similar sequences as if they were entirely distinct. In viral evolution, where small genetic changes can lead to significant phenotypic differences, this oversight could undermine our understanding of how viral populations adapt, evolve, and respond to selective pressures such as immune escape or antiviral treatments.

### 4.2 Similarity-weighted entropy metrics (*H_sim_* and *H_nsim_*): a more comprehensive measure of diversity

The introduction of similarity-weighted entropy metrics, *H*_sim_ and *H_n_*_sim_, addresses the limitations of traditional entropy by accounting for genetic similarity in addition to sequence frequency. As shown in Fig. 1, these metrics offer a more dynamic and comprehensive representation of viral quasispecies diversity. Weighted entropy has been applied in financial contexts, such as portfolio selection, to improve the accuracy of risk measures. Unlike standard entropy, which overlooks the spread of security frequency classes, weighting the entropy by state-value enhances the measure’s relevance. Tests using a portfolio selection algorithm show that this weighted approach improves investment performance (Nawrocki and Harding 1986).

In Figs 2 and 3, *H*_sim_ and *H_n_*_sim_ demonstrated sensitivity to both the frequency distribution of variants and the genetic similarity between them. As sequence similarity increased, these metrics decreased in value, indicating a reduced overall diversity within the population. This is particularly evident in Distribution 3 (Fig. 2c), where all sequences had equal frequency but different levels of sequence similarity. While *H* and *H_n_* remained constant across similarity levels, *H*_sim_ and *H_n_*_sim_ showed a clear decline in diversity as similarity increased, reflecting the fact that high sequence similarity reduces the effective genetic diversity of a viral population. This dynamic response illustrates that *H*_sim_ and *H_n_*_sim_ are better suited to populations with high genetic relatedness, a common feature of viral quasispecies, where a majority of variants are genetically similar to the master sequence.

The introduction of normalization in *H_n_*_sim_, which scales the value between 0 and 1, further enhances the sensitivity of the metric by making it comparable across populations with different levels of sequence richness and evenness. This normalization allows for more standardized comparisons of diversity across viral populations, improving its utility for understanding quasispecies evolution and adaptation.

### 4.3 Mean pairwise divergence: complementary but limited

The limitations of mean pairwise divergence as standalone measures of diversity are evident, even though the specific values for these indices were not provided. Mean pairwise divergence, which measures the average genetic distance between all pairs of sequences, provides valuable information about sequence dissimilarity but does not take into account the frequency or functional relevance of specific mutations. As shown in Fig. 3, mean pairwise divergence values remained constant across all nine distribution patterns, despite changes in sequence distribution. This highlights the metric’s inability to reflect how the distribution of variants within the population influences overall diversity. Moreover, as the number of sequences increases, mean pairwise divergence can provide skewed results, especially if there are many closely related variants. A large dataset might show a low divergence even if it contains significant functional diversity.

Therefore, mean pairwise divergence offers complementary insights into the genetic variation within viral populations, but it does not fully capture the complexity of diversity, especially in populations with high sequence similarity where the impact of genetic distance is more subtle. In contrast, *H*_sim_ and *H_n_*_sim_ integrate both the frequency and similarity of sequences, providing a more comprehensive and holistic measure of diversity.

### 4.4 Real-world application: viroid and virus quasispecies and functional diversity

In this study, the four entropy indices were applied to a real viroid quasispecies dataset, specifically the PSTVd. The analysis of 256 mutant sequences of a specific RNA loop revealed how variations in sequence similarity affect functional diversity. Using the similarity-weighted entropy metrics (*H*_sim_ and *H_n_*_sim_), I demonstrated that introducing variants with lower similarity to the master sequence could lead to the emergence of novel RNA structures, highlighting the functional implications of genetic diversity.

This real-world example illustrates that traditional entropy measures (*H* and *H_n_*) would not have captured the full extent of this functional diversity, as they do not account for the genetic similarity between the sequences. In contrast, *H*_sim_ and *H_n_*_sim_ provided a more comprehensive reflection of how changes in sequence similarity and distribution influence the genetic and functional diversity of the viral quasispecies. This finding underscores the importance of incorporating genetic similarity into diversity metrics, especially when studying populations where small genetic changes can lead to significant functional consequences, such as altered RNA structures and viral adaptability.

The analysis of the ToBRFV 5′-120nts quasispecies further highlights the advantages of similarity-weighted entropy over traditional metrics in assessing genetic diversity. Traditional metrics such as Hamming distance and Shannon entropy are commonly used together to evaluate viral quasispecies diversity. However, these metrics often focus on different aspects—sequence distribution versus sequence similarity—and can yield contradictory results. For instance, in this study, while the Hamming distance suggested higher genetic diversity in the *Tm-2^2^* sample, traditional Shannon entropy indicated the opposite. This inconsistency underscores the limitations of these traditional metrics in accurately capturing the complexities of viral populations. In contrast, the similarity-weighted entropy metrics, *H*_sim_ and *H_n_*_sim_, which incorporate both sequence distribution and similarity, provided a more nuanced understanding of genetic diversity. These metrics revealed the strong selection pressure exerted by the *Tm-2^2^* resistance allele on ToBRFV, highlighting the utility of similarity-weighted entropy in more accurately capturing viral quasispecies dynamics and analysing the role of resistance genes in shaping the structure of viral quasispecies.

## 5. Conclusion

This study demonstrates that the incorporation of sequence similarity into entropy calculations provides a more comprehensive measure of genetic diversity in viral quasispecies. The traditional entropy metrics (*H* and *H_n_*) are valuable for assessing population diversity in cases where sequence relatedness is low, but they fail to capture the subtleties of genetic diversity in populations with high sequence similarity. The similarity-weighted entropy indices, *H*_sim_ and *H_n_*_sim_, overcome this limitation by considering both sequence frequency and genetic similarity, offering a more nuanced and comprehensive representation of diversity. The real-world application to PSTVd viroid populations further supports the utility of these metrics in understanding the functional and genetic diversity of viral populations. As such, *H*_sim_ and *H_n_*_sim_ represent valuable tools for future studies of viral evolution, adaptation, and resistance, providing a more refined approach to analysing viral quasispecies and their potential for functional diversification. This enhanced understanding can inform the development of antiviral strategies, aiding in the prediction of novel viral variants and assessing the safety and efficacy of antiviral treatments.

## Supplementary Material

veaf029_Supp

## Data Availability

All the data are presented in this paper.
